# Prior childbirth experience and attitude towards subsequent vaginal birth after one caesarean delivery in Lagos, Nigeria: a cross-sectional study

**DOI:** 10.1186/s12884-023-05348-4

**Published:** 2023-01-30

**Authors:** Fatimat M. Akinlusi, Abideen A. Olayiwola, Kabiru A. Rabiu, Yusuf A. Oshodi, Tawaqualit A. Ottun, Khadijah A. Shittu

**Affiliations:** 1 Department of Obstetrics and Gynaecology, Lagos State University College of Medicine, Lagos, Nigeria; 2 Department of Obstetrics and Gynaecology, Lagos State University Teaching Hospital, No 1 – 5, Oba Akinjobi Way, Ikeja, Nigeria; 3 Department of Obstetrics and Gynaecology, Royal United Hospital, NHS Foundation Trust, Combe Park, Bath, BA1 3NG England

**Keywords:** Caesarean section, Prior caesarean delivery, Childbirth experience, Delivery intention

## Abstract

**Background:**

Prior caesarean delivery (CD) impacts CD rates in many parts of the world. In low and middle-income countries, few women attempt a trial of labour after caesarean delivery (TOLAC) due to inadequate resources for safe vaginal birth after caesarean delivery (VBAC). The CD rates continue to rise as more women undergo repeat CD. In Nigeria, VBAC rate is low and the contribution of women’s prior childbirth experiences and delivery wishes to this situation deserves further investigation. This study examined the parturient factor in the low VBAC rate to recommend strategies for change.

**Objective:**

To describe prior caesarean-related childbirth experiences and attitudes towards subsequent vaginal birth in pregnant women with one previous CD.

**Method:**

This cross-sectional study of antenatal clinic attendees in a tertiary hospital employed the convenience sampling method to recruit 216 consenting women with one previous CD. Structured questionnaires were used to collect information on participants' prior caesarean-related birth experiences, attitudes to vaginal birth in the index pregnancy, future delivery intentions and eventual delivery route. Univariate and bivariate analyses compared delivery wishes based on CD type. SPSS version 22.0 was used for data analysis.

**Results:**

The modal maternal and gestational age groups were 30–39 years (68.1%) and 29–34 weeks (49.1%) respectively; majorities (60.6%) were secundigravida; 61.6% experienced labour before their CDs while 76.9% had emergency CDs. Complications were documented in 1.4% and 11.1% of mothers and babies respectively. Ninety percent reported a satisfactory overall childbirth experience. A majority (83.3%) preferred TOLAC in the index pregnancy because they desired natural childbirth while 16.7% wanted a repeat CD due to the fear of fetal-maternal complications. The previous CD type and desire for more babies were significantly associated with respondents' preferred mode of delivery (*p* = 0.001 and 0.023 respectively). Women with previous emergency CD were more likely to prefer vaginal delivery.

**Conclusions:**

Antenatal women prefer TOLAC in subsequent pregnancies despite prior satisfactory caesarean-related birth experiences. Adoption of TOLAC in appropriately selected cases will impact women's psyche positively and reduce CD rate.

## Plain language summary

Prior caesarean delivery(CD) contributes to high CD rates especially in regions with limited medical infrastructures; as there is a quick resort to repeat CD. This study examined pregnant women’s previous caesarean birth experience and the impact on their choice of route for their next childbirth. Women were also interrogated on the number of children desired and their preferred intervals between future childbirths. Though, a majority reported prior satisfactory caesarean birth experience, four in five women would prefer vaginal birth to CD in the ongoing pregnancy; mostly because of their wish for natural childbirth. Women who experienced emergency caesarean births and those desirous of a higher number of babies were more likely to prefer vaginal birth. Therefore, we recommend that women (suitable for vaginal birth after one CD) be supported in their choice of vaginal delivery to enhance their psychological well-being, and reduce the need for repeat CD.

## Background

Prior caesarean delivery (CD) impacts CD rates in many parts of the world. Globally, the CD rate is high and increasing [1].An estimated 21·1% was recorded in 2015, almost double the proportion recorded in 2000 (12·1%) [1]. High CD rates of 25.7%, 39.3%, and up to 42.4% have been reported in Europe, the Americas and some Nigerian tertiary facilities respectively [1–4]. The proportion of women attempting a trial of labour after one previous caesarean delivery (TOLAC) is relatively low and decreasing [5, 6], especially in low and middle-income countries [7, 8], and this contributes to high CD rates [6]. The implication is that CD rates will continue to rise with more women having repeat CD rather than vaginal birth after cesarean delivery (VBAC) [6]. Decisive strategies need to be put in place to mitigate this.

For the woman who has had a prior CD, a successful trial of labour culminating in vaginal birth; a failed trial resulting in an emergency repeat CD; or an elective repeat CD are the three possible outcomes. Whichever approach is adopted, women who have undergone a prior CD should be informed about the maternal and neonatal risks and benefits associated with both planned VBAC and elective repeat caesarean delivery (ERCD) and those without contraindications to VBAC should be given an informed choice about the planned mode of birth after a previous CD [6, 9, 10].

Though regarded as a procedure with low risk, CD has intrinsic risks as studies indicate a 3 times higher risk of maternal death when compared to vaginal delivery[11] and 50 times as much risk of maternal mortality in certain African countries than in high-income countries [12].

Risks may be related to its indication and may predispose to complications such as puerperal infection, haemorrhage, thromboembolism and anaesthetic complications. Furthermore, future pregnancies are associated with an increased risk of various complications such as the increased risk of placenta praevia, morbidly adherent placentation, and surgical complications such as hysterectomy, especially in repeated ERCD [14]. Maternal morbidity increases with each additional caesarean section, especially for women with three or more caesarean sections who have a high risk of low insertion of the placenta, placenta accreta and hysterectomy [7]. Regarding fetal complications, the CD can also lead to increased iatrogenic prematurity and neonatal respiratory distress rates when performed without appropriate justification [14].

Though planned VBAC compared to ERCD is associated with a lower risk of maternal mortality, a shorter length of hospital stay, and a higher likelihood of breastfeeding, there is an increased risk of serious maternal complications such as uterine rupture, as well as a higher risk of perinatal/neonatal mortality and some types of neonatal morbidity [13]. Other risks include those of blood transfusion, puerperal sepsis, surgical injury as well as an increased risk of adverse perinatal outcomes as documented in a population-based cohort study of women with term singleton pregnancies and no contraindications to VBAC where planned VBAC was compared to ERCD [15]. TOLAC complications arise mainly from the need for emergency repeat CD in cases in which VBAC has not been achieved [16]. These complications can be minimized with good patient selection by identifying parturient likely to achieve a successful VBAC and those not [17]. Appropriate intrapartum monitoring is also imperative.

Several factors such as the client's acceptability of TOLAC, Obstetricians' willingness [18, 19] to offer it and the facility available affect the adoption of TOLAC. Although not all women with prior CD may be eligible for TOLAC; in well-selected cases, as many as 75% have successful vaginal delivery [6].

Despite abounding evidence that clients' attitudes may be contributory to the uptake of VBAC, the client factor has not been extensively explored. It is well-known that experiences that emerged in prior childbirth may be related to acceptance or refusal of a particular mode of delivery [20, 21].

At the Lagos State University Teaching Hospital, Ikeja, the CD rate is alarming at 40%. We investigated prior childbirth experiences; delivery route preferences and future reproductive plans of pregnant women with one previous CD. This group serves as a major reservoir for repeat CD and may play a significant role in the rising incidence of CD. We also highlight factors associated with preference for subsequent vaginal delivery after one previous CD.

## Method

### Study design and setting

This questionnaire-based cross-sectional descriptive study, with longitudinal follow-up, was conducted at the maternity outposts of the Lagos State University Teaching hospital (LASUTH), Nigeria, between 1st April and 30th September 2017.

Lagos is an urban settlement with a population of approximately 18 million; there are 20 local governments and 37 local council development areas [22]. It is one of the most populous and fastest-growing cities in the world. A significant proportion of the population is educated. The consultant-led antenatal clinics ran twice weekly at each of the maternity outposts of Ifako-Ijaye and Isolo. Averages of five new clients register per clinic day while about forty attend for follow-up.

### Participants

These were antenatal clinic attendees, who had experienced only one previous caesarean delivery irrespective of gestational age, pregnancy risks, gravidity or parity.

### Inclusion and exclusion criteria

All antenatal clinic attendees with one previous CD were informed about the study and the opportunity to enroll. All consenting pregnant women were included irrespective of the inter pregnancy interval. Pregnant women with more than one CD; those with no antecedent history of CD and non-consenting women were excluded.

### Sample size determination and sampling

An a priori sample size calculation established a sample of 216 participants using a prevalence of 50% for previous good childbirth experience; 5.0% error margin; with 10% added for attrition. Convenience sampling was used for recruitment.

### Study tool and outcomes measurement

The study tool was a structured interviewer-administered questionnaire that was developed by the research team from previous studies. It was pilot-tested on 20 antenatal women with prior CD to assess its clarity. Feedback and analysis from this process were used to improve the questionnaire before conducting the study. Face validity of the tool was done by two Obstetricians. Sections addressed respondents' socio-demographics; childbirth experiences around the previous CD; and delivery intentions. Trained House Officers who were fluent in the local language doubled as interviewers and interpreters in collecting data from eligible and consenting pregnant women.

The primary outcome was the participant's previous caesarean-related childbirth experience. Our secondary outcomes included attitude towards vaginal delivery in the index pregnancy and other delivery wishes. Explanatory variables such as CD type, experience of pain and labour were assessed. Post-delivery, we ascertained the eventual mode of delivery via phone calls to mothers, two weeks after their expected date of delivery (EDD). Case records were reviewed for the delivery mode for respondents who could not be reached on phone.

### Statistical analysis

Participants’ characteristics were expressed as absolute and relative frequencies (categorical data) or as means and standard deviations (numeric data). Chi-squared test or Fischer’s exact test (used when > 20% of the expected frequencies are < 5) were used to compare the association between previous CD type and prior childbirth experience, preferred MOD and future delivery intentions. The level of significance was set at a *p*-value < 0.05. Statistical Product and Service Solutions (SPSS) version 22 was used for all statistical analysis.

### Ethical consideration

The Health Research and Ethics Committee of Lagos State University Teaching Hospital granted institutional review board approval; Ref No; LREC.06/10/1038. Written informed consent was obtained from all women for their participation in the study.

## Results

Overall, 216 antenatal patients were interviewed. The response rate was 100% as data were complete in all the questionnaires administered. Table 1 shows the socio-demographic characteristics of the respondents. About two-thirds (68.1%) of respondents were aged 30-39 years; the mean age was 32.5 ± 4.5 years. Majorities (88.9%) were Christians; 69.9% were Yoruba while 77.3% had tertiary level education; 60.6% were in their second pregnancies and almost half (49.1%) were between 29–34 weeks of gestation.

**Table 1:** Socio-demographic characteristics of respondents

Variable	Frequency (*n* = 216)	Percentage (%)
**Age group (years)**		
20–29	56	25.9
30–39	147	68.1
≥ 40	13	6.0
Mean SD	32.51 ± 4.5	
**Religion**		
Christianity	192	88.9
Islam	24	11.1
**Ethnic group**		
Yoruba	151	69.9
Igbo	46	21.3
Others	19	8.8
**Highest educational level**		
Primary	5	2.3
Secondary	44	20.4
Tertiary	167	77.3
**Gravidity**		
2	131	60.6
3	58	26.9
4	15	6.9
> 4	12	5.6
**Number of children**		
1	183	84.7
2	30	13.9
3	2	0.9
4	1	0.5
**Gestational age**		
≤ 28	75	34.7
29–34	106	49.1
≥ 35	35	16.2

Three in four participants (76.9%) had emergency CD. The association between socio-demographic characteristics and the previous CD type is depicted in Table 2. There were no significant associations between respondents' age, gravidity, parity, gestational age and CD type (*p* = 0.350; 0.447, 0.091 and 0.888) respectively).

**Table 2:** Association between socio-demographic characteristics and CD type

	**Prior CD Type**		**Total**	***P *value**
	**Elective (*n* = 50)**	**Emergency (*n* = 166)**		
**Age group (years)**				
20–29	11(19.6)	45(80.4)	56(100.0)	*p* = 0.350*
30–39	34(23.1)	113(76.9)	147(100.0)	
≥ 40	5(38.5)	8(61.5)	13(100.0)	
**Gravidity**				
2	28(21.4)	103(78.6)	131(100.0)	*p* = 0.0447*
3	14(24.1)	44(75.9)	58(100.0)	
4	3(20.0)	12(80.0)	15(100.0)	
> 4	5(41.7)	7(58.3)	12(100.0)	
**Number of children**				
1	38(20.8)	145(79.2)	183(100.0)	*p* = 0.091**
2	10(33.3)	20(66.7)	30(100.0)	
3	1(50.0)	1(50.0)	2(100.0)	
4	1(100.0)	0(0.0)	1(100.0)	
**Gestational age**				
≤ 28	18(24.0)	57(76.0)	75(100.0)	*p* = 0.888*
29–34	25(23.6)	81(76.4)	106(100.0)	
≥ 35	7(20.0)	28(80.0)	35(100.0)	

*chi-square test, **Fisher's exact test

Table 3 shows respondents' caesarean-related childbirth experiences. About two thirds (61.6%) of the respondents experienced labour before the CD; 28.2% experienced some degree of pain during surgery; while post-operative pain was moderate and severe in 42.6% and 13.0% respectively. Twenty-four babies (11.1%) and three mothers (1.4%) developed complications. Birth asphyxia, cerebral palsy, infection and death occurred in 62.5%, 8.3%, 4.2% and 25% respectively. Respondents' overall experience of childbirth was satisfactory in majorities (90.7%) of respondents.

**Table 3:** Previous childbirth experience

Variable	Frequency (*n* = 216)	Percentage
**Labour before CD**		
Yes	133	61.6
No	83	38.4
**Experience of pain during surgery**		
None	155	71.8
Mild	45	20.8
Moderate	14	6.5
Severe	2	0.9
**Experience of pain after surgery**		
None	48	22.2
Mild	48	22.2
Moderate	92	42.6
Severe	28	13.0
**Maternal complication**		
Yes	3	1.4
No	213	98.6
**Neonatal complication**		
Yes	24	11.1
No	192	88.9
**Complication type (*n* = 24)**		
Birth asphyxia	15	62.5
Infection	1	4.2
Cerebral palsy	2	8.3
Death	6	25.0
**Overall experience of childbirth**		
Satisfactory	196	90.7
Unsatisfactory	20	9.3

Table 4 shows the association between the CD type and childbirth experience. The CD type was significantly associated with prior experience of labour; postoperative pain and neonatal complication (*p* < 0.001; *p* = 0.037 and *p* = 0.024 respectively). However, there were no significant associations between CD type and experience of pain during surgery, maternal complication after surgery and respondents' overall experience of childbirth (*p* = 0.075), (*p* = 0.674) and (*p* = 0.726) respectively. Respondents who had emergency CD were more likely to experience labour, postoperative pain and neonatal complications, though the CD type did not impact the overall childbirth experience.

**Table 4:** Association between prior childbirth experience and CD Type

	**Prior Caesarean type**		**Total**	**Statistics**
	**Elective (*n* = 50)**	**Emergency (*n *= 166)**		
**Labour prior to CD**				
Yes	0(0.0)	133(100.0)	133(100.0	*p* < 0.001**
No	50(60.2)	33(39.8)	83(100.0)	
**Intra-operative pain level**				
None	43(27.7)	112(72.3)	155(100.0)	*p* = 0.075**
Mild	6(13.3)	39(86.7)	45(100.0)	
Moderate	1(7.1)	13(92.9)	14(100.0)	
Severe	0(0.0)	2(100.0)	2(100.0)	
**Post-operative pain level**				
None	18(37.5)	30(62.5)	48(100.0)	*p* = 0.037*
Mild	12(25.0)	36(75.0)	48(100.0)	
Moderate	15(16.3)	77(83.7)	92(100.0)	
Severe	5(17.9)	23(82.1)	28(100.0)	
**Maternal complication**				
Yes	1(33.3)	2(66.7)	3(100.0)	*p* = 0.674**
No	49(23.0)	164(77.0)	213(100.0)	
**Neonatal complication**				
Yes	5(20.8)	19(79.2)	24(100.0	*p* = 0.776*
No	45(23.4)	147(76.6)	192(100.0)	
**Baby complication (*n* = 24)**				
Birth asphyxia	3(20.0)	12(80.0)	15(100.0)	*p* = 0.024**
Infection	0(0.0)	1(100.0)	1(100.0)	
Cerebral palsy	2(100.0)	0(0.0)	2(100.0)	
Death	0(0.0)	6(100.0)	6(100.0)	
**Overall experience**				
Satisfactory	46(23.5)	150(76.5)	196(100.0)	*p* = 0.726**
Unsatisfactory	4(20.0)	16(80.0)	20(100.0)	

^*^chi-square test, ^**^Fisher's exact test, significant *P* values are shown in bold

Women who had previous emergency CD preferred to have a vaginal delivery in the ongoing pregnancy **(***p* < 0.001) and were more likely to desire more babies (*p* = 0.023) than those who had elective CD (Table 5).

**Table 5:** Association between maternal delivery intention and type of CD

	**Elective (*n* = 50)**	**Emergency (*n* = 166)**	**Total**	**Statistics**
**Preferred delivery**				
Vaginal delivery(VD)	33(18.3)	147(81.7)	180(100.0)	*p* < 0.001*
Caesarean delivery(CD)	17(47.2)	19(52.8)	36(100.0)	
**Reason for VD (*n* = 180**				
Desire it	28(18.5)	123(81.5)	151(100.0)	*p* = 0.544**
Fear of Pain with CD	3(27.2)	8(72.7)	11(100.0)	
Cost of CD	2(11.1)	16(88.9)	18(100.0)	
**Reason for CD (*n* = 36)**				
Fear of labour	4(40.0)	6(60.0)	10(100.0)	*p* = 0.039**
Poor labour experience	0(0.0)	5(100.0)	5(100.0)	
Fear of complication	13(61.9)	8(38.1)	21(100.0)	
**Desire more babies**				
Yes	34(20.4)	133(79.6)	167(100.0)	*p* = 0.023*
No	16(32.7)	33(67.3)	49(100.0)	
**No desired (*n* = 167)**				
One	26(23.0)	87(77.0)	113(100.0)	*p* = 0.219*
Two	8(14.8)	46(85.2)	54(100.0)	
**Year desired(*n*** **= 167)**				
2	23(23.2)	76(76.8)	99(100.0)	*p* = 0.538**
3	8(16.0)	42(84.0)	50(100.0)	
> 3	3(16.7)	15(83.3)	18(100.0)	
**Why more(*n*** **= 49)**				
Health reason	3(60.0)	2(40.0)	5(100.0)	*p* = 0.159**
Social reason	10(37.0)	17(63.0)	27(100.0)	
Financial reason	3(17.6)	14(82.4)	17(100.0)	

^*^chi-square test, ^**^Fisher's exact test, significant *P* values are shown in bold

However, there were no significant associations between prior CD type and reasons for vaginal delivery preference; the number of additional babies desired; timing of the next baby and respondents' plan not to have more babies (*p* = 0.544), (*p* = 0.219), (*p* = 0.538) and (*p* = 0.159).

**Fig. 1:**
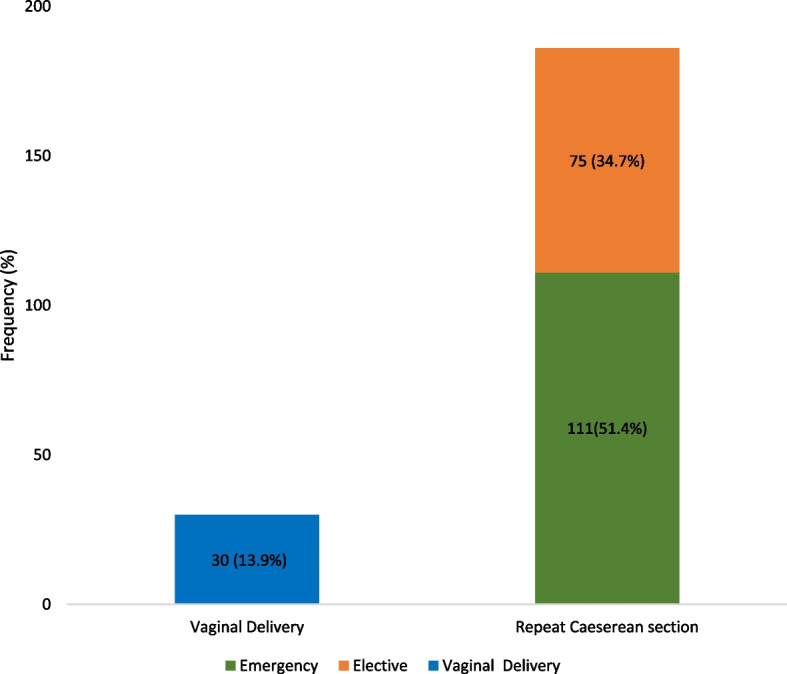
Eventual Mode of Delivery of respondents with one previous caesarean delivery. The Bar Chart depicts the delivery routes; 51.4% and 34.7% were via emergency and elective CD respectively; 13.9% had successful vaginal births after CD

At post-partum follow-up, the eventual mode of delivery was via emergency CD in 51.4% of respondents; by elective CD in 34.7% while 13.9% of them had successful vaginal births after one previous caesarean delivery as shown in Fig. 1.

## Discussion

Our study explored prior childbirth experience and maternal attitude towards subsequent vaginal birth in antenatal women with one previous CD. Though a majority (90.7%) of the respondents reported satisfactory overall experience in their previous caesarean-related childbirth experience, a high percentage (83.3%) still expressed a preference for vaginal birth in the index pregnancy. All participants desired more children after the index pregnancy. This is not unexpected as the fertility rate in our environment is high.

The mean age of 32.51 ± 4.5 years is comparable to 30.2 and 32.11 ± 4.2 years reported by Olofinbiyi and Phocas Biraboneye et al., [20, 23]. All were married which is comparable to the Kenyatta study where 94% of respondents were married [23]. A higher proportion of our respondents (77.7%) had tertiary education, comparable to that reported by Garmaroudi et al [24], unlike the Kenyatta study where just 45.6% had secondary education [23]. Our study population is from the high-literacy urban region of Lagos. In terms of respondents' previous delivery, 84.7% of respondents had one previous delivery of a live baby, 13.9% had two, while 0.9% and 0.5% of them had three and four deliveries respectively. This is comparable to that reported in the Kenyatta study in which 75.7% of respondents had previous delivery of one life baby [23].

More than a quarter (28.2%) of our respondents and 14% (7/50) of the elective CD group experienced intra-operative pain. This exceeds the 5% standard set for intra-operative pain experience in elective patients by the Royal College of Anaesthetist [25]. Post-operative pain was reported as moderate and severe by 42.6% and 13.0% of our respondents respectively. An Oslo study also reported inadequate post-operative pain relief in 68% of their respondents [26].

It is well known that the majority of CDs are performed under neuraxial anaesthesia [27] which often provides adequate analgesia. Yet, a small proportion may feel pain which may require conversion to general anaesthesia [25]. Maternal intra-operative experience of pain may be due to failed spinal anaesthesia and reluctance to convert an inadequate regional anesthesia to general anesthesia. Failure to convert an existing labour epidural analgesia to epidural anesthesia for CD may be contributory and the reported incidence ranges from 1.7% to 19.8% [28].

Acute post-surgical pain contributes to the development of chronic post-surgical pain in women who have undergone CD [29].

Our maternal complication rate in the previous CD was much lower than the 15.3% reported by Phocas Biraboneye et al [23]. Neonatal death accounted for 25% of all neonatal complications in our study, which is less than 39.5% reported in a similar study [23]. This variation may be due to differences in the resources available at the healthcare facilities. Maternal morbidity increases with additional CDs, especially in women with three or more CD who have a high risk of low insertion of the placenta, placenta accreta and hysterectomy [30].

The majority of our respondents (83.3%) prefer to have a trial of labour after CD mostly because of their wish to experience the natural route of vaginal delivery (83.9%). Our findings are comparable to those reported by Olofinbiyi et al. and Onah et al. where a considerable proportion of their respondents declined a repeat CD [20, 31].

Olofinbiyi reported that about 69.2% of their respondents would accept a repeat CD if medically indicated, while the remaining 38.2% would not accept [20]. Parity, maternal educational status, number of previous CDs and outcomes of previous deliveries did not show a significant association with acceptance or refusal of repeat CD. Refusal of CD was due to religious belief, fear of surgical pain, desire for vaginal delivery, cost of surgery, stress of surgery, fear of death and postoperative scar [20].

Maternal preferences have significant impact on decisions about the route of delivery. Our findings, that the majority of women with one previous CD prefer vaginal birth subsequently, might enhance positive discussions between obstetricians and clients regarding the delivery route. The implication is that these women are more likely to be offered TOLAC by their obstetricians which may translate into increased VBAC rates. Interventions to reduce CD rates may need to target healthcare providers and health facilities rather than pregnant women.

In women with a previous transverse lower segment CD, a trial of labour after CD is a reasonable option [6, 10, 20]. Previous studies show that both labour and elective CD in pregnant women with one previous CD are associated with significant risks and benefits, which differ for the mother and the fetus (risk of uterine rupture, febrile morbidity, need for blood transfusion and hysterectomy) [32].

Characteristics that are associated with a favourable outcome of a TOLAC are a non-recurring indication for the first CD and a previous history of vaginal delivery in multiparous women with a previous CD [31]. It is of note that all women with a prior CD may not be eligible for a trial of vaginal delivery, and even when selected for a vaginal birth after caesarean delivery (VBAC), vaginal delivery may not be successful in about 23.5% of women undergoing a TOLAC [23].

Phocas Biraboneye et al. documented a preference for elective repeat caesarean delivery(ERCD) in 67.2% of respondents [23]. Their study population probably dreaded the risks associated with a TOLAC and perhaps had a poor knowledge of the success rate associated with TOLAC. Olofinbiyi et al. also reported that 30.8% of their respondents would refuse a repeat CD [20].

In our society, people believe that women who have not experienced vaginal birth cannot be considered as having reproductive capability [23]. Of the 16.7% of respondents who desired a repeat CD, 58.3% of them preferred it because of fear of maternal or fetal complication. All participants documented their intention to have one or two more babies, after the index pregnancy.

Comparable to earlier studies, the sociodemographic factors did not impact previous CD type [20].

Prior CD type impacted maternal intention to have more babies (*p* = 0.023) and attitude to vaginal birth (*p* < 0.001) which is comparable to findings in a similar study [23]. However, the CD type was not significantly associated with the number of babies, birth interval, reason for more babies (*p* = 0.219), (*p* = 0.538) and (*p* = 0.159) respectively.

Despite the preference for vaginal delivery in 83.3% of the respondents, at post-partum follow-up, only 13.9% had a successful VBAC, while 34.7% and 51.4% had elective and emergency CD respectively. Literature has documented various reasons why eligible patients end up having repeat CD. The avoidance of litigation; mothers’ preference for planned labour-free delivery; and the assumption that CD prevents delivery complications have been cited [33]. Subjective rather than objective clinical indications such as fetal distress and labour-arrest disorders [34]; the counseling given by healthcare providers, who are guided primarily by their opinions about repeat CD and TOLAC [35] are additional reasons. There seems to be a rising inclination of obstetricians to tow the CD route and mothers have reported experiencing pressure from health professionals to have a CD [36].

Our study did not explore the reasons for this outcome. However, it will pave the way for further local longitudinal studies that will explore why patients end up with repeat CD.

### Study strength and limitation

Our focus on women's mode of birth preferences is in tandem with a women-centred approach to care. However, the views of healthcare providers were not explored in this instance. Childbirth experiences are predisposed to recall bias, the cross-sectional design cannot ascertain the causal relationship between the outcome and explanatory variables and it is institutional-based.

## Conclusion

Our study revealed that most antenatal women with one previous CD had satisfactory caesarean-related childbirth experience. However, a considerable proportion would prefer a TOLAC mostly because they desire it. The majority preferred to have at least one more child within the succeeding two years. Most women with previous CD wish to have an average of three children.

Knowledge of women’s preference for vaginal birth may encourage obstetricians to offer TOLAC more frequently to eligible women. Doing so will not only delight the women but also go a long way in increasing VBAC rate in our centre. This may help women with previous CD realize their future delivery intentions. Prospective, multi-centre studies are desirable to further explore this topic and identify significant predictors to enable the development of strategies to increase VBAC rates.

## Data Availability

The datasets used for this study will be available from the corresponding author on reasonable request and in accordance with consent and ethical approval.

## Abbreviations

CD: Caesarean delivery
EDD: Expected date of delivery
ERCD: Elective repeat caesarean delivery
LASUTH: Lagos State University Teaching Hospital
TOLAC: Trial of labour after caesarean delivery
VBAC: Vaginal birth after caesarean delivery
